# Type-IVC Secretion System: A Novel Subclass of Type IV Secretion System (T4SS) Common Existing in Gram-Positive Genus *Streptococcus*

**DOI:** 10.1371/journal.pone.0046390

**Published:** 2012-10-04

**Authors:** Wen Zhang, Chengbo Rong, Chen Chen, George F. Gao

**Affiliations:** 1 National Institute for Communicable Disease Control and Prevention, Chinese Center for Disease Control and Prevention/State Key Laboratory for Infectious Disease Prevention and Control, Beijing, China; 2 CAS Key Laboratory of Pathogenic Microbiology and Immunology, Institute of Microbiology, Chinese Academy of Science, Beijing, China; 3 Beijing Institutes of Life Science, Chinese Academy of Sciences, Beijing, China; University of Iowa Carver College of Medicine, United States of America

## Abstract

A growing number of pathogens are being found to possess specialized secretion systems which they use in various ways to subvert host defenses. Type IV secretion system (T4SS) is one of versatile secretion systems essential for the virulence and even survival of some bacteria species, and they enable the secretion of protein and DNA substrates across the cell envelope. T4SS was once believed to be present only in Gram-negative bacteria. In this study, we present evidence of a new subclass of T4SS, Type-IVC secretion system and indicate its common existence in the Gram-positive bacterial genus *Streptococcus.* We further identified that VirB1, VirB4, VirB6 and VirD4 are the minimal key components of this system. Using genome comparisons and evolutionary relationship analysis, we proposed that Type-IVC secretion system is movable via transposon factors and mediates the conjugative transfer of DNA, enhances bacterial pathogenicity, and could cause large-scale outbreaks of infections in humans.

## Introduction

A growing number of pathogens are being found to possess specialized secretion systems which they use in various ways to subvert host defenses [1]. Type IV secretion system (T4SS) is one of versatile secretion systems essential for the virulence and even survival of some bacteria species, and they enable the secretion of protein and DNA substrates across the cell envelope [2], [3], [4], [5].

Based on a number of characteristics, including, the organization of genetic determinants, shared homologies and evolutionary relationships, T4SSs have been divided into several subgroups: Type-IVA, Type-IVB systems and other T4SSs [6], [7]. The *Agrobacterium tumefaciens* Vir system is considered to be the paradigm of Type-IVA, which consists of 12 components, VirB1−VirB11 and VirD4 [7]. VirB1, with its lytic transglycosylase subunits, can create holes in the cell wall to enable the movement of T4SS [3], [8]. VirB2 and VirB5 are pilus components [3], [9], [10]. Three cytoplasmic ATPases, VirB4, VirB11, and VirD4, provide energy for substrate secretion and assist in the assembly of the T4SS [3]. VirD4 is referred to as a coupling protein which recruit the substrates to the T4SS for translocation [11], [12]. VirB6 and VirB8 are polytopic inner membrane proteins essential for substrate secretion through the inner membrane in Gram-negative bacteria [3], [13], while VirB7-VirB9-VirB10 forms a stable core complex that spans the cell membrane [3], [14]. The type-IVB secretion system was initially found in *Legionella pneumophila* and is composed of 25 genes on two separate regions. Region I contains seven genes (*icmV, W and X, and dotA, B, C* and *D*), and Region II contain the other 18 genes (*icmT, S, R, Q, P, O, N, M, L, K, E, G, C, D, J, B, F* and *H*) [15]. The majority of these genes were also found in the genome sequences of *Coxiella burnetti* [*15]*, [16]. More recently, a novel lineage of T4SSs classified as “others” have been identified on the genomic island ICE*Hin1056* of *Haemophilus influenza* [7], [17].

T4SS was once believed to be present only in Gram-negative bacteria but has been found in Gram-positive organisms as well. Our previous work support that in Gram-positive species of *Streptococcus suis* (*S. suis*), a GI-type T4SS-like system was identified in a new pathogenicity island (PAI) with a length of 89 kb [18], which was further proven to be a new subgroup of T4SS in this study. *S. suis*, a Gram-positive species of *Streptococcus* found in pigs, has recently caused a rash of human infections in China and gained public attention [19], [20], [21], [22]. In these epidemic *S. suis* isolates related to two recent large-scale outbreaks of human infection in China, we identified this new 89 kb PAI with GI-type T4SS-like system [18]. Our works also showed that this 89K PAI can spontaneously excise to form an extrachromosomal circular product, laterally conjugally transfer to non-89K *S. suis* recipients through the 89K-encoded GI-type T4SS [23]. Based on genome comparisons and evolution relationship analysis in the current study, we identified that this GI-type T4SS-like system is a new subgroup T4SS named as Type-IVC secretion system for its clearly different genetic organization with type-IVA and type-IVB in Gram-negative bacteria. VirB1, VirB4, VirB6 and VirD4 were proven to be the minimal key component of type-IVC secretion system. We further identified that type-IVC secretion system is unexpectedly popular in the genus *Streptococcus*. This system is movable with the help of transposon factors (such as Tn916), which could also mediate the conjugative transfer of DNAs and enhance bacterial pathogenicity.

## Materials and Methods

### 1. Genome Sequences of *Streptococcus*

The *Streptococcus* strains used in the current study are listed in Supplemental Tables. Whole genome sequences of 50 strains of *Streptococcus* (Table 1) were downloaded from the NCBI ftp (ftp://ftp.ncbi.nih.gov/genomes). Draft genome sequences of 67 *Streptococcus* strains were also obtained from the NCBI database (Table S2).

**Table 1:** List of *Streptococcus* strains with whole genomes used in the current study.

Species	Strain Number	Strain Name
*Streptococcus agalactiae*	3	*Streptococcus agalactiae* 2603V R uid57943
		*Streptococcus agalactiae* A909 uid57935
		*Streptococcus agalactiae* NEM316 uid61585
*Streptococcus dysgalactiae*	1	*Streptococcus dysgalactiae* equisimilis GGS 124 uid59103
*Streptococcus equi*	3	*Streptococcus equi* 4047 uid59259
		*Streptococcus equi* zooepidemicus MGCS10565 uid59263
		*Streptococcus equi* zooepidemicus uid59261
*Streptococcus gallolyticus*	2	*Streptococcus gallolyticus* ATCC BAA 2069 uid63617
		*Streptococcus gallolyticus* UCN34 uid46061
*Streptococcus gordonii*	1	*Streptococcus gordonii* Challis substr CH1 uid57667
*Streptococcus mitis*	1	*Streptococcus mitis* B6 uid46097
*Streptococcus mutans*	2	*Streptococcus mutans* NN2025 uid46353
		*Streptococcus mutans* UA159 uid57947
*Streptococcus pneumoniae*	14	*Streptococcus pneumoniae* 670 6B uid52533
		*Streptococcus pneumoniae* 70585 uid59125
		*Streptococcus pneumoniae* AP200 uid52453
		*Streptococcus pneumoniae* ATCC 700669 uid59287
		*Streptococcus pneumoniae* CGSP14 uid59181
		*Streptococcus pneumoniae* D39 uid58581
		*Streptococcus pneumoniae* G54 uid59167
		*Streptococcus pneumoniae* Hungary19A 6 uid59117
		*Streptococcus pneumoniae* JJA uid59121
		*Streptococcus pneumoniae* P1031 uid59123
		*Streptococcus pneumoniae* R6 uid57859
		*Streptococcus pneumoniae* Taiwan19F 14 uid59119
		*Streptococcus pneumoniae* TCH8431 19A uid49735
		*Streptococcus pneumoniae* TIGR4 uid57857
*Streptococcus pyogenes*	13	*Streptococcus pyogenes* M1 GAS uid57845
		*Streptococcus pyogenes* Manfredo uid57847
		*Streptococcus pyogenes* MGAS10270 uid58571
		*Streptococcus pyogenes* MGAS10394 uid58105
		*Streptococcus pyogenes* MGAS10750 uid58575
		*Streptococcus pyogenes* MGAS2096 uid58573
		*Streptococcus pyogenes* MGAS315 uid57911
		*Streptococcus pyogenes* MGAS5005 uid58337
		*Streptococcus pyogenes* MGAS6180 uid58335
		*Streptococcus pyogenes* MGAS8232 uid57871
		*Streptococcus pyogenes* MGAS9429 uid58569
		*Streptococcus pyogenes* NZ131 uid59035
		*Streptococcus pyogenes* SSI 1 uid57895
*Streptococcus sanguinis*	1	*Streptococcus sanguinis* SK36 uid58381
*Streptococcus suis*	5	*Streptococcus suis* 05ZYH33 uid58663
		*Streptococcus suis* 98HAH33 uid58665
		*Streptococcus suis* BM407 uid59321
		*Streptococcus suis* P1/7 uid32235
		*Streptococcus suis* SC84 uid59323
*Streptococcus thermophilus*	3	*Streptococcus thermophilus* CNRZ1066 uid58221
		Streptococcus thermophilus LMD 9 uid58327
		*Streptococcus thermophilus* LMG 18311 uid58219
*Streptococcus uberis*	1	*Streptococcus uberis* 0140J uid57959

We manually collected 839 gene sequences of the 12 components of T4SS (*virB1*−*virB11* and *virD4*) from the NCBI and UniprotKB database [24], [25].

### 2. Genome-wide Search for T4SS Genes and *virB*/*D* Cluster

To search for genes in the T4SS, a new program (TFSP) was used (http://t4ss.bioinfo-icdc.org/). This program combines the alignment algorithm approaches, prediction of protein functions, and domain evaluation to detect candidate T4SS genes (*virB1*−11 and *virD4*) in genomes of bacteria (the detailed method is shown in the Protocol S1) with good precision.

Based on the locations of identified T4SS genes (*virB*/*D* genes), we found *virB*/*D* clusters in genomes of *Streptococcus*. In this study, determination of *virB*/*D* clusters conformed to the following criteria: (1) the distance between two nearby *virB*/*D* genes is less than 5 kb, (2) the total length of the *virB*/*D* cluster is less than 50 kb, and (3) the number of *virB*/*D* genes in a *virB*/*D* cluster is ≥3.

### 3. Identification of Genomic Islands (GI) with *virB*/*D* Cluster

For co-lineage comparisons, Blast similarity searches were performed using local BLAST software [26]. Based on the Blast results, the GI and its precise location in the genome were determined by the co-lineage comparison between a genome with a *virB*/*D* cluster and one without it [27]. The GC content skew of GI was step-analyzed in a window of 2,000 bp by a self-developed Perl program (draw_GC_content.pl) [27]. The function of genes in the GI was annotated by the method of Clusters of Orthologous (COG) [28].

### 4. Construction of Phylogenetic Tree

*rpoB* is a housekeeping gene and highly conserved in many bacteria. We used *rpoB* gene sequences in 50 species to generate a phylogenetic tree of *Streptococcus*. Multiple sequence alignments of these gene sequences were performed using MEGA [29]. A phylogenetic tree was constructed using the neighbor-joining algorithm in Mega, and 1,000 subsets were generated for bootstrapping re-sampling of the data. Another tree was built based on the concatenated sequences of *virB4*, *virB6*, and *virD4* genes using the same method.

## Results

### A New Subgroup of T4SS in Gram-positive *S. suis:* Type-IVC Secretion System

**Figure 1:**
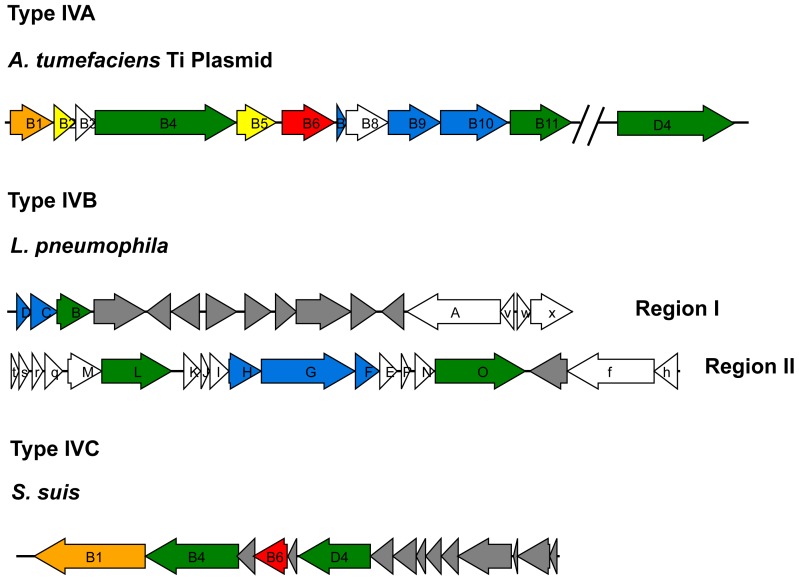
Genetic organization of T4SS. For *A. tumefaciens*, gene names B = *virB* and D4 = *virD4*. For *L. pneumophila,* upper-case gene names = *dot* and lower-case gene names = *icm*. Genes in orange correspondents to periplasmic lytic transglycosylase, in green ATPase, in yellow T pilus, in red channel subunit across inner membrane, in blue channel subunit across outer membrane, in grey no T4SS genes or genes with the unclear function.

**Figure 2:**
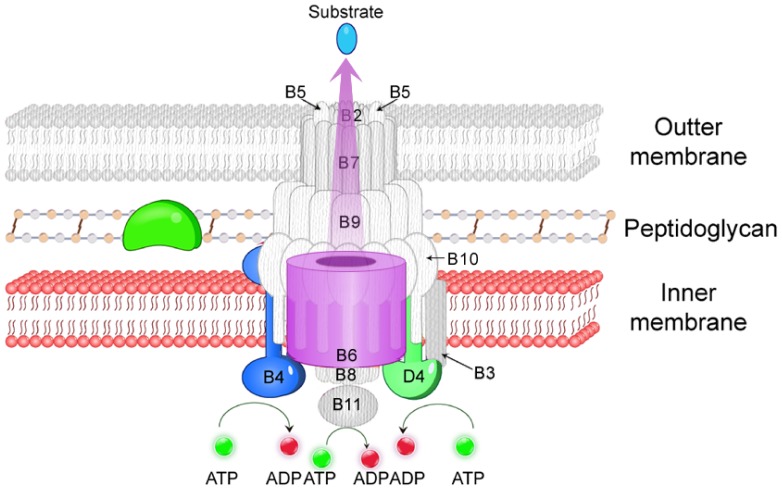
Hypothetical model for Type-IVC secretion system. The colored parts are the components of the Type-IVC secretion system system. The grey parts are objects present in the canonical T4SS of Gram-negative strains but lost in the Gram-positive Type-IVC secretion system, e.g., the outer membrane, VirB2, and VirB5.

A new subgroup of T4SS (GI-type T4SS) was identified in Gram-positive strain *S. suis* [18], [23]. Genetic organization represented that this GI-type T4SS in Gram-positive strain *S. suis* is clearly different with type-IVA, type-IVB and other T4SS in Gram-negative bacteria, thus it is classified as Type-IVC secretion system in this study (Figure 1). Different with other T4SS systems, only 4 proteins (VirB1, VirB4, VirB6, and VirD4) were identified in Type-IVC secretion systems, which mainly work in three fields: (1) transglycosylases (VirB1), working for degrading peptidoglycan outside the plasma membrane of bacteria, could reduce the resistance for the secretion of substrates; (2) ATPases (VirB4, and VirD4) play essential roles in supplying the energy for substrates translocation and apparatus assembly [4]; (3) a gene contributing to the assembly of the secretion channel across inner cell membrane(VirB6). These genes clustered together with the same direction in the chromosome. Those genes correspondent to channel subunit across outer membrane, such as VirB7/VirB9/VirB10 in type-IVA and DotD/DotC/DotH/DotG/DotF in type-IVB, were lost in type-IVC secretion system (Figure 1 and Figure 2).

### Popular Existence of Type-IVC Secretion System in Genus *Streptococcus*

To more fully understand the distribution of Type-IVC secretion system in genus *Streptococcus*, we used bioinformatic methods to predict T4SS genes (Figure S2) in 50 *Streptococcus* strains with published genome sequences (Table 1). Detailed methods are described in the Method section. 15 Type-IVC secretion systems were identified in 14 of 50 *Streptococcus* strains (Table S1). *S. suis* BM407 (NC_012926) has two copies of T4SS, while the other 13 genomes have only one. All 15 Type-IVC secretion systems own clustered *virB1-like/virB4/virB6/virD4* or *virB4/virB6/virD4* genes in the same direction and order (Table S1 and Figure S2). The Type-IVC secretion system in *S. pyogenes* MGAS2096 also has homolog of *virB2* gene. Our genome analysis represent that, most strains in *Streptococcus* were identified with *virB/D* genes (Figure S1) and approximately 28% of all *Streptococcus* strains have Type-IVC secretion system (Table S1).

Additional investigations into the 67 draft genomes of *Streptococcus* strains (Table S2) further supported the belief that Type-IVC secretion system are popular in *Streptococcus*; in these studies, 19 Type-IVC secretion system were identified (Table S2).

### Evolutionary Relationship Among Type-IVC Secretion Systems in Genus *Streptococcus*

Among 50 strains of *Streptococcus*, 14 strains own additional DNA fragments (GI, 50–89 kb) with Type-IVC secretion system by co-lineage comparisons genomic comparison. Compared with the 89 kb PAI of *S. suis*, the structures of these GIs are significantly variable (Figure S2): Their locations in the genomes are different and genes in these GIs are also variable. However, they retain some common features. First, all the GIs have *virB/D* gene clusters with similar genes. Second, genes in the *virB/D* clusters always have the same order and direction. Third, most of the clusters own a transposon, such as Tn916.

**Figure 3:**
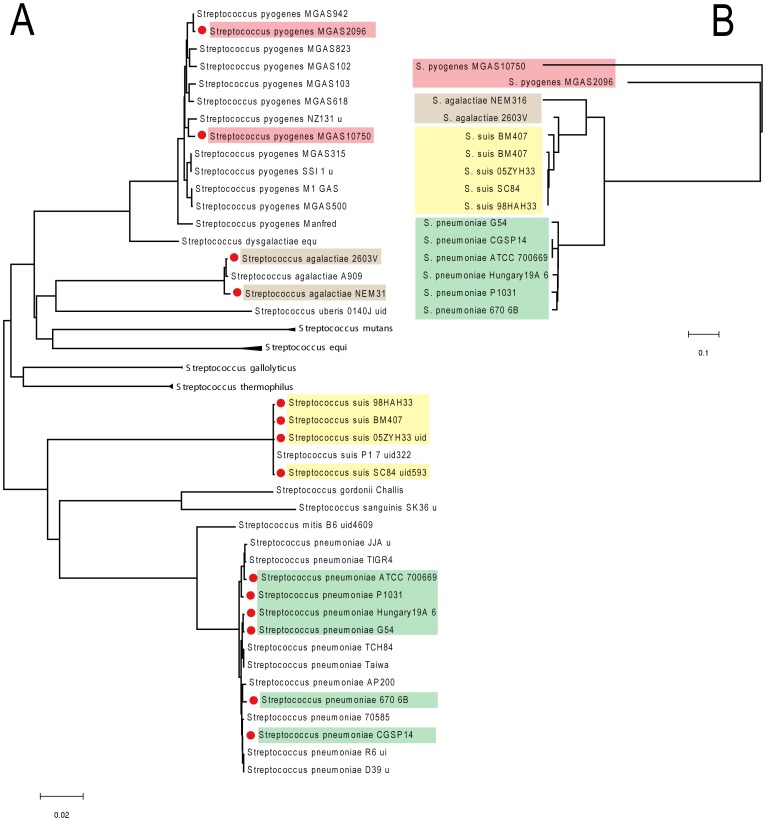
The phylogenetic tree. (A) The neighbor-joining (NJ) tree obtained on the basis of the housekeeping gene *rpoB*. The red solid circles represent GIs with *virB/D* clusters. (B) NJ tree obtained on the basis of a concatenated sequence of *virB4*, *virB6*, and *virD4* genes in GI.

The phylogenetic tree obtained from the housekeeping gene *rpoB* clearly showed that Type-IVC secretion system could be present or absent in different strains of a species (Figure 3A). The occurrence of Type-IVC secretion system in these branches could not be caused by a mutation, but could perhaps be caused by several rounds of DNA acquisition from other strains. Our previous experiment proved that a GI with Type-IVC secretion system can spontaneously excise to form an extrachromosomal circular product and laterally transfer to another strain [23]. Considering the proven mobility of Type-IVC secretion system, we believe that Horizontal Transfers (HT) between species brought about the acquisition of Type-IVC secretion system in *Streptococcus*. Figure 3B further shows that *virB/D* genes in Type-IVC secretion system have higher similarity in the same species than that between two species, suggesting that the movement of GIs caused by HT occurs more easily within strains of a species than that between species.

## Discussion

### Minimal Components of Type-IVC Secretion System

In Gram-negative bacteria, Type-IVA secretion system usually consists of 12 components to work together just as the figure 2 shown. Thorough investigations about functions of these 12 components showed that these genes mainly work in three fields: (1) transglycosylases (such as VirB1), working for degrading peptidoglycan outside the plasma membrane of bacteria, could reduce the resistance for the secretion of substrates; (2) ATPases (VirB4, VirD4 and VirB11) play essential roles in supplying the energy for substrates translocation and apparatus assembly [4]; (3) these are still some genes contributing to the assembly of the secretion channel across inner (VirB6 and VirB8) and outer membranes (VirB7, VirB9 and VirB10).

In Type-IVC secretion system of *Streptococcus*, although only four components were identified (VirB1, VirB4, VirB6, and VirD4), they still work in same three fields (Figure 2). VirB1-like protein, with CHAP domain, could encode amidohydrolases and is responsible for punching holes through the peptidoglycan outside of the gram-positive cell membrane. VirB4 and VirD4 could supply energizes necessary for the substrate transport [4]. VirB6, the inner-membrane protein, is to compose the transport channel across the cell membrane. Since the lack of the outer membrane in Gram-positive bacteria, it is not a surprise that proteins contributing to the assembly of the secretion channel across outer membranes (VirB7, VirB9 and VirB10) in type-IVA secretion systems have not been found in Type-IVC secretion system. The other components in 89K PAI may also play role in substrates transport but the determination of their functions requires further investigation. We propose that VirB1, VirB4, VirB6 and VirD4 are the minimal key component of Type-IVC secretion system (Figure 2).

The function of these four proteins (VirB1, VirB4, VirB6, and VirD4) for the secretion of DNA has been proven by making individual knockouts of T4SS gene homologues *(ΔvirB1-89K, ΔvirB4-89K, ΔvirB6-89K and ΔvirD4-89K*) [23]. Our previous results showed that no transconjugants were obtained when *ΔvirB4-89K, ΔvirB6-89K* and *ΔvirD4-89K* were used as donors. And the transfer frequency of *ΔvirB1-89K* (9.2×10^−7^) was six-fold less than that of wild type strains [23]. Our other experiments in experimental infection of mice showed that knockout of VirB4 and VirD4 in this Type-IVC secretion system also eliminated the lethality of the highly virulent strain and impaired its ability to trigger host immune response [30].

### Mobility of Type-IVC Secretion System

The phylogenetic tree of the housekeeping gene *rpoB* clearly showed that Type-IVC secretion system is movable, for strains with and without Type-IVC secretion system co-existing in several branches (Figure 3A). The popular existence of Type-IVC secretion system could not be only caused by mutations. HT between species underlie the presence of GIs with T4SS in *Streptococcus*. Figure 3B further indicates that the movement of type-IVC secretion system occurs more easily within the strains of a species than that between species.

Our previous experimental work also proved that type-IVC secretion system was movable. In *S. suis* 05ZYH33, 89-kb GI that could spontaneously excise to form an extrachromosomal circular product and lateral transfer from a donor to a recipient cell at a frequency of 10^−6^ transconjugants/donor [23].

The HT of type-IVC secretion system is under the help of transposons. In the nearby DNA sequences of 15 Type-IVC secretion system in *Streptococcus sp.*, 9 of 15 (60%) were identified with Tn916 transposons (Figure S2). Although without Tn916 nearby Type-IVC secretion systems, two strains (*S. pneumoniae CGSP14* and *S. pyogenes MGAS2096)* harbored Tn916 residues, whereas the other two strains (*S. pneumoniae Hungary19A and S. agalactiae 2603V*) had Tn916 at another position on the chromosome (Figure S2). Of the 4 strains without Tn916, 3 were found to have other transposons, like Tn5252 and Tn5421. Considering the surprisingly high correlation between transposons and Type-IVC secretion system, we could reasonably assume that some mobile elements necessary for the HT of T4SS were introduced by transposons. These mobile elements help T4SS move between cells, and the T4SS enables transposons to cross the barriers between donor and recipient cells. We believe that a random confusion between a transposon and minimal T4SS led to the formation of Type-IVC secretion system. Type-IVC secretion system gradually accumulated in *Streptococcus* species by HT, and an increasing number of strains harbor this system. During transport, the loss or acquisition of variable elements in this Type-IVC secretion system is ongoing. Further experimental work is necessary to support our hypothesis and answer questions regarding when the random event leading to the formation of Type-IVC secretion system occurred and whether HT of this system occurs between different species.

### Function of Type-IVC Secretion System in Pathogenicity of *Streptococcus*

The Type-IVC secretion system could enhance bacterial pathogenicity and mediate the injection of virulent proteins into host cells. Our previous study showed that the two component system SalK/R within the 89-kb island controls the virulence of the highly pathogenic strain *S. suis* 2 [23]. Recently, using NimbleGen tiling arrays, Zhu and colleagues found this 89-kb fragment in 9 other virulent *S. suis* 2 lineages, all of which were sampled from two recent large-scale outbreaks of human infection in China [31]. The 89-kb GI which include Tn916 with tetracycline-resistance genes of the *S. suis* 2 strain was proved could laterally transfer to other *S. suis* 2 strains with the help of Type-IVC secretion system [23]. Knockout of the 2 key components (VirD4 and VirB4) of the *S. suis* 2 T4SS system eliminated the lethality of the highly virulent strain and impaired its ability to trigger host immune response in experimental infection of mice [30]. All of this evidence together suggests that Type-IVC secretion system contributes to the pathogenicity of *S. suis* 2, particularly in the two outbreaks of *S. suis* 2 infections in China.

### Conclusions

In this paper, we present evidence that the GI-type T4SS-like system we experimentally defined earlier in *S. suis* is unexpectedly popular in the genus *Streptococcus* based on an analysis of deposited genome sequences. It always located in a GI with abnormal GC content. VirB1, VirB4, VirB6 and VirD4 are the minimal key component of this system in *Streptococcus*. We propose that this system in Gram-positive bacteria is a new subclass of T4SS (Type-IVC secretion system). Further, it is movable with the help of transposon factors, which could mediate the conjugative transfer of plasmid DNA/transposons and enhance bacterial pathogenicity.

## Supporting Information

Figure S1 Strain numbers in which *virB1–virB11* and *virD4* genes were identified. The blue columns are the number of *Streptococcus* strains with *virB/D* genes, whereas the red columns are the number of *Streptococcus* strains with *virB/D* clusters.
(DOC)

Figure S2 Genome islands with T4SS in 10 strains of *Streptococcus* compared to the 89-kb GI in *S. suis* 05ZYH33. GC%, locations of the *virB/D* genes, Tn916, and other important genes in GI are shown. A) *S. suis SC84*; B) *S. suis BM407* (1000794∼1091159); C) *S. suis BM407* (499472∼585444); D) *S. pneumoniae P1031*; E) *S. pneumoniae G54*; F) *S. pneumoniae ATCC 700669*; G) *S. pneumoniae CGSP14*; H) *S. agalactiae 2603V R*; I) *S. agalactiae NEM316*; J) *S. pyogenes MGAS2096*. Genes with varying functions are presented in different colors.
(DOC)

Table S1 List of *virB/D* clusters identified in 14 *Streptococcus* strains. ID: *virB/D* cluster ID in a genome; S: start site of *virB/D* gene in genome; E: end site of *virB/D* gene in genome; D: direction of *virB/D* gene.
(DOC)

Table S2 List of *Streptococcus* strains with draft genomes used in this study. “+” indicates that there is an identified *virB/D* gene cluster in this strain.
(DOC)

Protocol S1 Detailed Material and Methods.
(DOC)
