# Supplementary material for: Type-IVC Secretion System: A Novel Subclass of Type IV Secretion System (T4SS) Common Existing in Gram-Positive Genus Streptococcus
Source: PLoS One. 2012 Oct 4;7(10):e46390. doi: 10.1371/journal.pone.0046390 (PMC3464263; doi:10.1371/journal.pone.0046390)

**Figure S1.** Strain numbers with identified *virB1*–*virB11*, and *virD4* genes. Blue columns are number of *Streptococcus* strains with *virB*/*D* genes, whereas red columns are number of *Streptococcus* strains with *virB*/*D* clusters.


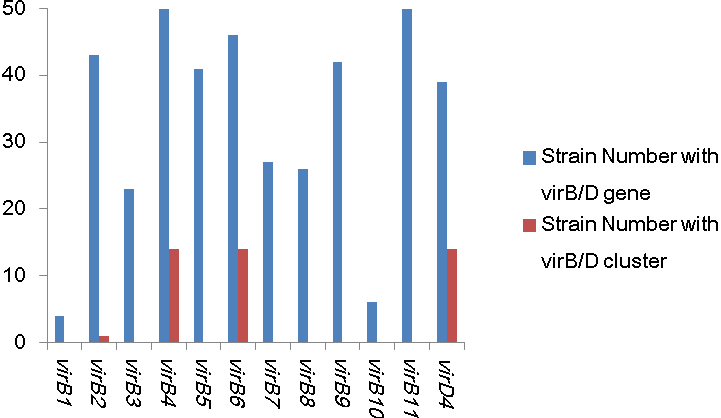

Supplement: Figure S1 — Strain numbers in which virB1–virB11 and virD4 genes were identified. The blue columns are the number of Streptococcus strains with virB/D genes, whereas the red columns are the number of Streptococcus strains with virB/D clusters. (DOC) [file pone.0046390.s001.doc]
