# Supplementary material for: Type-IVC Secretion System: A Novel Subclass of Type IV Secretion System (T4SS) Common Existing in Gram-Positive Genus Streptococcus
Source: PLoS One. 2012 Oct 4;7(10):e46390. doi: 10.1371/journal.pone.0046390 (PMC3464263; doi:10.1371/journal.pone.0046390)

**Figure S2.** Genome islands with T4SS in ten strains of *Streptococcus* comparing to 89K GI in *S.suis* 05ZYH33. Supplemental Figure showed GC% and locations of *virB*/*D* genes, Tn916 and other important genes in GI. A) *S. suis SC84*; B) *S. suis BM407* (1000794~1091159); C) *S. suis BM407* (499472~585444); D) *S. pneumonia P1031*; E) *S. pneumonia G54*; F) *S. pneumonia ATCC 700669*; G) *S. pneumonia CGSP14*; H) *S. agalactiae 2603V R*; I) *S. agalactiae NEM316*; J) *S. pyogenes MGAS2096*; K) *S. suis* 98HAH33; L) *S. pneumonia* 670 6B; M) *S. pneumonia* Hungary19A 6; N) *S. pyogenes* MGAS10750.Description of the genes with various functions is presented in each color.


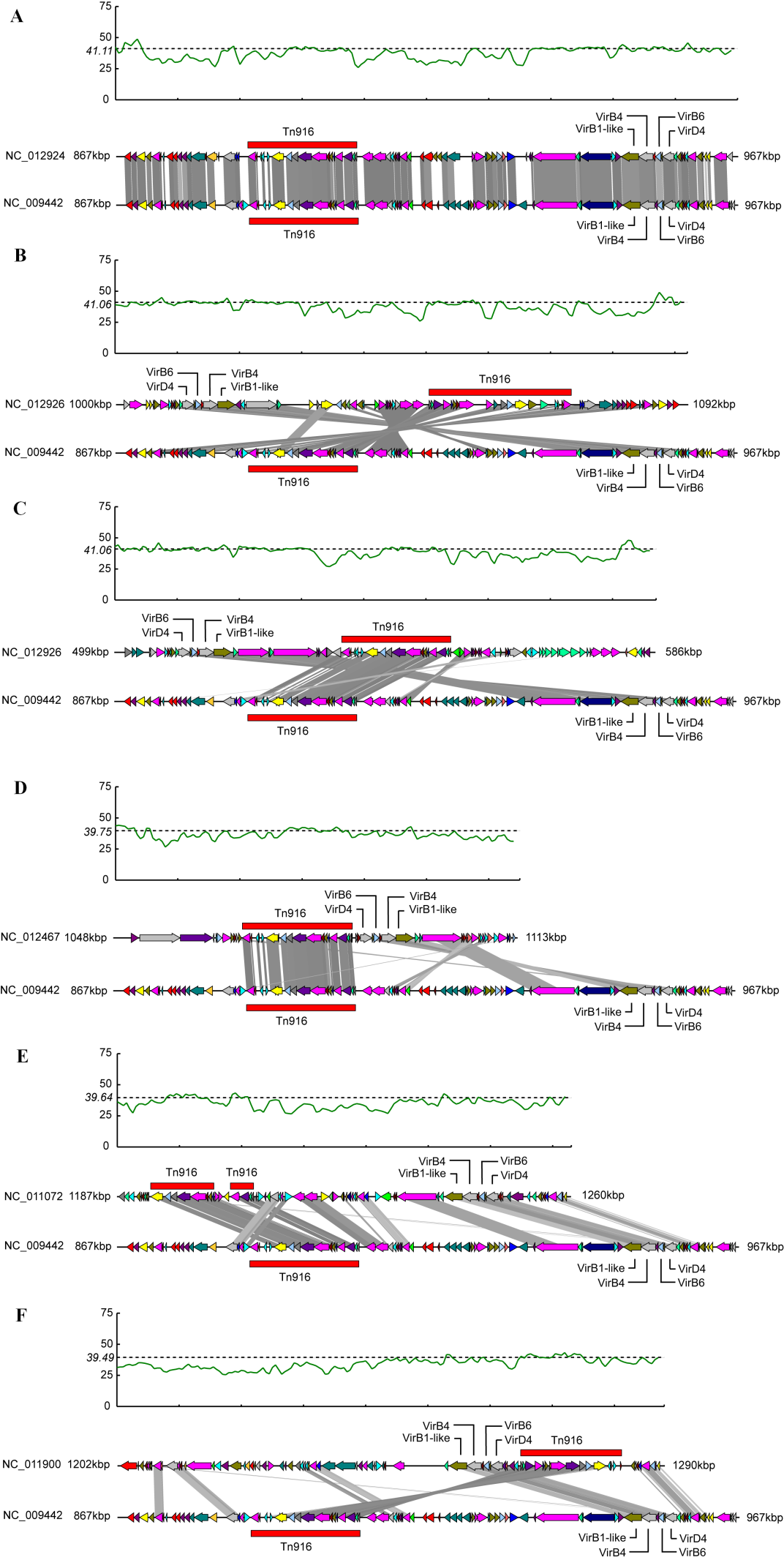


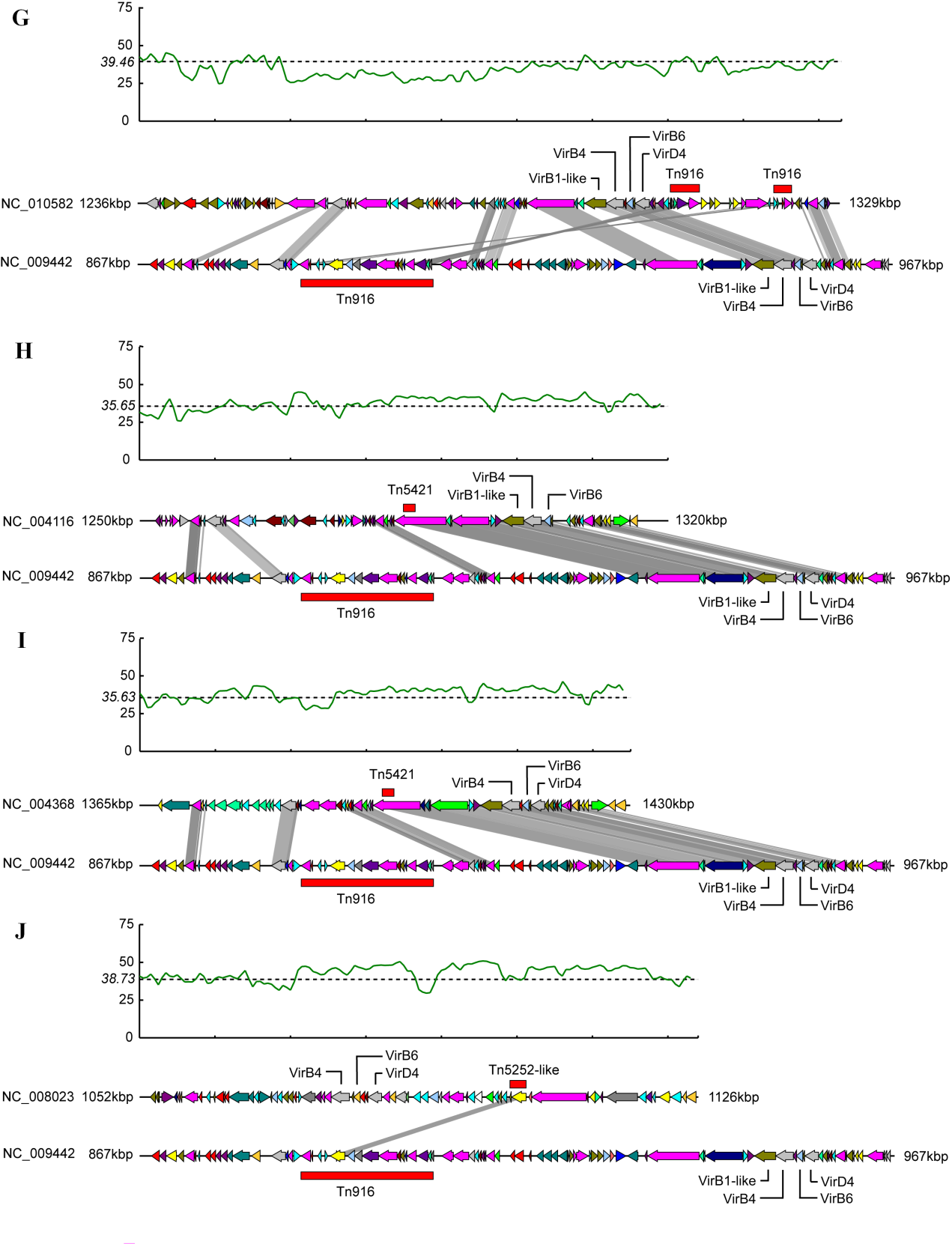

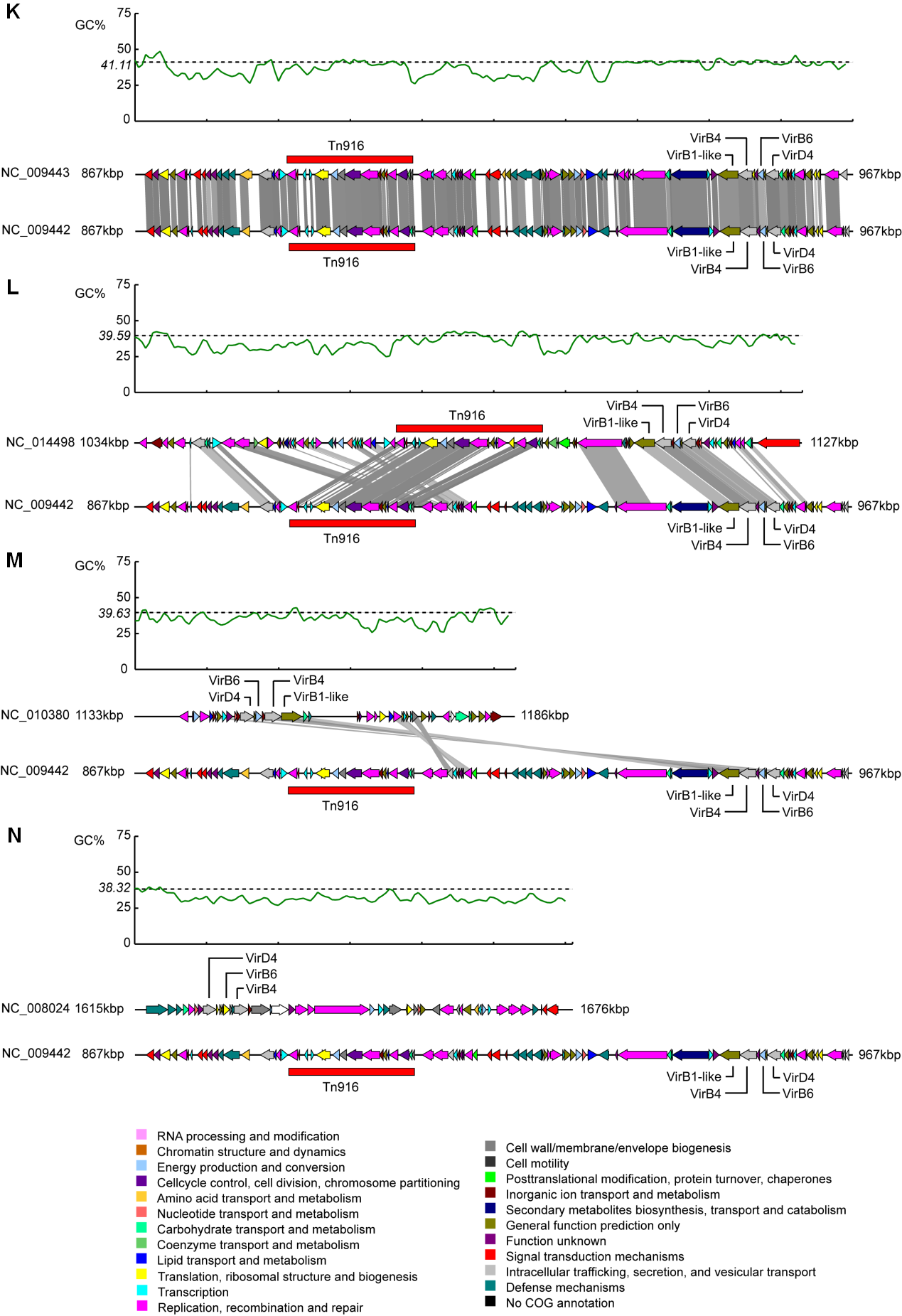

Supplement: Figure S2 — Genome islands with T4SS in 10 strains of Streptococcus compared to the 89-kb GI in S. suis 05ZYH33. GC%, locations of the virB/D genes, Tn916, and other important genes in GI are shown. A) S. suis SC84; B) S. suis BM407 (1000794∼1091159); C) S. suis BM407 (499472∼585444); D) S. pneumoniae P1031; E) S. pneumoniae G54; F) S. pneumoniae ATCC 700669; G) S. pneumoniae CGSP14; H) S. agalactiae 2603V R; I) S. agalactiae NEM316; J) S. pyogenes MGAS2096. Genes with varying functions are presented in different colors. (DOC) [file pone.0046390.s002.doc]
