# Supplementary material for: Type-IVC Secretion System: A Novel Subclass of Type IV Secretion System (T4SS) Common Existing in Gram-Positive Genus Streptococcus
Source: PLoS One. 2012 Oct 4;7(10):e46390. doi: 10.1371/journal.pone.0046390 (PMC3464263; doi:10.1371/journal.pone.0046390)
Supplement: Table S1 — List of virB/D clusters identified in 14 Streptococcus strains. ID: virB/D cluster ID in a genome; S: start site of virB/D gene in genome; E: end site of virB/D gene in genome; D: direction of virB/D gene. (DOC) [file pone.0046390.s003.doc]

**Table S1.** List of *virB/D* clusters identified in 14 genomes of Genus *Streptococcus*. ID: *virB/D* cluster ID in a genome; S: start site of *virB*/*D* gene in genome; E: end site of *virB*/*D* gene in genome; D: direction of *virB*/*D* gene.

| **Genome ID** | **Strain Name** | **ID** | **Name of *virB*/*D* genes in Cluster** | **Type** | **S** | **E** | **D** |
| --- | --- | --- | --- | --- | --- | --- | --- |
| NC_004368 | *Streptococcus agalactiae* NEM316 uid61585 | 1 | gbs1359 | *virB1-like* | 1410217 | 1413012 | – |
|  |  |  | gbs1360 | *virB4* | 1413014 | 1415359 | – |
|  |  |  | gbs1362 | *virB6* | 1415731 | 1416585 | – |
|  |  |  | gbs1364 | *virD4* | 1416864 | 1418681 | – |
| NC_004116 | *Streptococcus agalactiae* 2603V R uid57943 | 1 | SAG1286 | *virB1-like* | 1298030 | 1300831 | – |
|  |  |  | SAG1287 | *virB4* | 1300833 | 1303163 | – |
|  |  |  | SAG1289 | *virB6* | 1303556 | 1304410 | – |
| NC_014498 | *Streptococcus pneumoniae* 670 6B uid52533 | 1 | SP670_1182 | *virB1-like* | 1103677 | 1106490 | – |
|  |  |  | SP670_1183 | *virB4* | 1106502 | 1108817 | – |
|  |  |  | SP670_1185 | *virB6* | 1109223 | 1110077 | – |
|  |  |  | SP670_1187 | *virD4* | 1110357 | 1112234 | – |
| NC_011900 | *Streptococcus pneumoniae* ATCC 700669 uid59287 | 1 | SPN23F_12840 | *virB1-like* | 1255346 | 1258159 | – |
|  |  |  | SPN23F_12850 | *virB4* | 1258171 | 1260486 | – |
|  |  |  | SPN23F_12870 | *virB6* | 1260892 | 1261746 | – |
|  |  |  | SPN23F_12890 | *virD4* | 1262026 | 1263906 | – |
| NC_010582 | *Streptococcus pneumoniae* CGSP14 uid59181 | 1 | SPCG_1308 | *virB1-like* | 1295217 | 1298030 | – |
|  |  |  | SPCG_1309 | *virB4* | 1298042 | 1300357 | – |
|  |  |  | SPCG_1311 | *virB6* | 1300763 | 1301617 | – |
|  |  |  | SPCG_1313 | *virD4* | 1301897 | 1303777 | – |
| NC_011072 | *Streptococcus pneumoniae* G54 uid59167 | 1 | SPG_1275 | *virB1-like* | 1239715 | 1242528 | – |
|  |  |  | SPG_1276 | *virB4* | 1242540 | 1244897 | – |
|  |  |  | SPG_1278 | *virB6* | 1245261 | 1246115 | – |
|  |  |  | SPG_1280 | *virD4* | 1246395 | 1248272 | – |
| NC_010380 | *Streptococcus pneumoniae* Hungary19A 6 uid59117 | 1 | SPH_1242 | *virD4* | 1147760 | 1149637 | + |
|  |  |  | SPH_1244 | *virB6* | 1149917 | 1150771 | + |
|  |  |  | SPH_1246 | *virB4* | 1151177 | 1153492 | + |
|  |  |  | SPH_1247 | *virB1-like* | 1153504 | 1156317 | + |
| NC_012467 | *Streptococcus pneumoniae* P1031 uid59123 | 1 | SPP_1171 | *virD4* | 1087730 | 1089607 | + |
|  |  |  | SPP_1173 | *virB6* | 1089887 | 1090741 | + |
|  |  |  | SPP_1175 | *virB4* | 1091147 | 1093462 | + |
|  |  |  | SPP_1176 | *virB1-like* | 1093474 | 1096287 | + |
| NC_008024 | *Streptococcus pyogenes* MGAS10750 uid58575 | 1 | MGAS10750_Spy1683 | *virD4* | 1624512 | 1626323 | + |
|  |  |  | MGAS10750_Spy1687 | *virB6* | 1627347 | 1628210 | + |
|  |  |  | MGAS10750_Spy1690 | *virB4* | 1628813 | 1630831 | + |
| NC_008023 | *Streptococcus pyogenes* MGAS2096 uid58573 | 1 | MGAS2096_Spy1125 | *virB4* | 1077345 | 1079774 | – |
|  |  |  | MGAS2096_Spy1127 | *virB6* | 1080367 | 1081218 | – |
|  |  |  | MGAS2096_Spy1129 | *virB2* | 1081864 | 1082235 | – |
|  |  |  | MGAS2096_Spy1130 | *virD4* | 1082250 | 1084043 | – |
| NC_009442 | *Streptococcus suis* 05ZYH33 uid58663 | 1 | SSU05_0968 | *virB1-like* | 948449 | 951250 | – |
|  |  |  | SSU05_0969 | *virB4* | 951252 | 953603 | – |
|  |  |  | SSU05_0971 | *virB6* | 953975 | 954829 | – |
|  |  |  | SSU05_0973 | *virD4* | 955109 | 956926 | – |
| NC_009443 | *Streptococcus suis* 98HAH33 uid58665 | 1 | SSU98_0981 | *virB1-like* | 948153 | 950954 | – |
|  |  |  | SSU98_0982 | *virB4* | 950956 | 952929 | – |
|  |  |  | SSU98_0985 | *virB6* | 953678 | 954532 | – |
|  |  |  | SSU98_0987 | *virD4* | 954811 | 956628 | – |
| NC_012926 | *Streptococcus suis* BM407 uid59321 | 1 | SSUBM407_0464 | *virD4* | 509342 | 511159 | + |
|  |  |  | SSUBM407_0466 | *virB6* | 511438 | 512292 | + |
|  |  |  | SSUBM407_0468 | *virB4* | 512685 | 515015 | + |
|  |  |  | SSUBM407_0469 | *virB1-like* | 515017 | 517818 | + |
|  |  | 2 | SSUBM407_0941 | *virD4* | 1010686 | 1012503 | + |
|  |  |  | SSUBM407_0943 | *virB6* | 1012782 | 1013636 | + |
|  |  |  | SSUBM407_0945 | *virB4* | 1014029 | 1016359 | + |
|  |  |  | SSUBM407_0946 | *virB1-like* | 1016361 | 1019162 | + |
| NC_012924 | *Streptococcus suis* SC84 uid59323 | 1 | SSUSC84_0876 | *virB1-like* | 948193 | 950994 | – |
|  |  |  | SSUSC84_0877 | *virB4* | 950996 | 953326 | – |
|  |  |  | SSUSC84_0879 | *virB6* | 953719 | 954573 | – |
|  |  |  | SSUSC84_0881 | *virD4* | 954852 | 956669 | – |
