# Supplementary material for: Type-IVC Secretion System: A Novel Subclass of Type IV Secretion System (T4SS) Common Existing in Gram-Positive Genus Streptococcus
Source: PLoS One. 2012 Oct 4;7(10):e46390. doi: 10.1371/journal.pone.0046390 (PMC3464263; doi:10.1371/journal.pone.0046390)
Supplement: Table S2 — List of Streptococcus strains with draft genomes used in this study. “+” indicates that there is an identified virB/D gene cluster in this strain. (DOC) [file pone.0046390.s004.doc]

**Table S2.** List of *Streptococcus* strains with draft genomes used in this study. “+” represent that there is identified *virB*/*D* gene cluster in this strain.

| **Species** | **Strain Num** | **Strain Name** | **VirB/D cluster** |
| --- | --- | --- | --- |
| *Streptococcus* *sp*. | 1 | *Streptococcus* 2 1 36FAA uid41507 | + |
| *Streptococcus agalactiae* | 5 | *Streptococcus agalactiae* 18RS21 uid54309 | + |
|  |  | *Streptococcus agalactiae* 515 uid54311 | + |
|  |  | *Streptococcus agalactiae* CJB111 uid54313 |  |
|  |  | *Streptococcus agalactiae* COH1 uid54317 | + |
|  |  | *Streptococcus agalactiae* H36B uid54315 | + |
| *Streptococcus anginosus* | 2 | *Streptococcus anginosus* 1 2 62CV uid62163 |  |
|  |  | *Streptococcus anginosus* F0211 uid61277 | ++ |
| *Streptococcus australis* | 1 | *Streptococcus australis* ATCC 700641 uid62169 |  |
| *Streptococcus bovis* | 1 | *Streptococcus bovis* ATCC 700338 uid52359 | + |
| *Streptococcus C150* | 1 | *Streptococcus* C150 uid62525 |  |
| *Streptococcus C300* | 1 | *Streptococcus* C300 uid62527 |  |
| *Streptococcus cristatus* | 1 | *Streptococcus cristatus* ATCC 51100 uid62537 | + |
| *Streptococcus downei* | 1 | *Streptococcus downei* F0415 uid60561 |  |
| *Streptococcus equinus* | 1 | *Streptococcus equinus* ATCC 9812 uid62297 |  |
| *Streptococcus gallolyticus* | 1 | *Streptococcus gallolyticus* TX20005 uid52357 |  |
| *Streptococcus infantarius* | 1 | *Streptococcus infantarius* ATCC BAA 102 uid54885 | + |
| *Streptococcus infantis* | 2 | *Streptococcus infantis* ATCC 700779 uid62539 |  |
|  |  | *Streptococcus infantis* SK1302 uid59469 |  |
| *Streptococcus M143* | 1 | *Streptococcus* M143 uid42367 |  |
| *Streptococcus M334* | 1 | *Streptococcus* M334 uid62529 | + |
| *Streptococcus mitis* | 5 | *Streptococcus mitis* ATCC 6249 uid52355 |  |
|  |  | *Streptococcus mitis* NCTC 12261 uid52969 |  |
|  |  | *Streptococcus mitis* SK321 uid52973 |  |
|  |  | *Streptococcus mitis* SK564 uid52971 |  |
|  |  | *Streptococcus mitis* SK597 uid52967 | + |
| *Streptococcus oralis* | 2 | *Streptococcus oralis* ATCC 35037 uid53033 | + |
|  |  | *Streptococcus oralis* uid47255 |  |
| *Streptococcus oral* | 1 | *Streptococcus oral* taxon 071 73H25AP uid52351 | + |
| *Streptococcus parasanguinis* | 3 | *Streptococcus parasanguinis* ATCC 15912 uid49313 |  |
|  |  | *Streptococcus parasanguinis* ATCC 903 uid62541 |  |
|  |  | *Streptococcus parasanguinis* F0405 uid60563 |  |
| *Streptococcus parauberis* | 1 | *Streptococcus parauberis* NCFD 2020 uid65825 | + |
| *Streptococcus peroris* | 1 | *Streptococcus peroris* ATCC 700780 uid62543 |  |
| *Streptococcus pneumoniae* | 24 | *Streptococcus pneumoniae* BS397 uid51551 |  |
|  |  | *Streptococcus pneumoniae* BS455 uid51543 |  |
|  |  | *Streptococcus pneumoniae* BS457 uid51553 |  |
|  |  | *Streptococcus pneumoniae* BS458 uid51555 |  |
|  |  | *Streptococcus pneumoniae* Canada MDR 19A uid42621 |  |
|  |  | *Streptococcus pneumoniae* Canada MDR 19F uid42623 |  |
|  |  | *Streptococcus pneumoniae* CCRI 1974M2 uid55647 |  |
|  |  | *Streptococcus pneumoniae* CCRI 1974 uid55563 | + |
|  |  | *Streptococcus pneumoniae* CDC0288 04 uid54893 |  |
|  |  | *Streptococcus pneumoniae* CDC1087 00 uid54923 |  |
|  |  | *Streptococcus pneumoniae* CDC1873 00 uid54921 |  |
|  |  | *Streptococcus pneumoniae* CDC3059 06 uid54891 |  |
|  |  | *Streptococcus pneumoniae* MLV 016 uid54929 |  |
|  |  | *Streptococcus pneumoniae* SP11 BS70 uid54499 |  |
|  |  | *Streptococcus pneumoniae* SP14 BS292 uid51549 |  |
|  |  | *Streptococcus pneumoniae* SP14 BS69 uid54501 |  |
|  |  | *Streptococcus pneumoniae* SP18 BS74 uid54503 |  |
|  |  | *Streptococcus pneumoniae* SP195 uid54889 |  |
|  |  | *Streptococcus pneumoniae* SP19 BS75 uid54505 |  |
|  |  | *Streptococcus pneumoniae* SP23 BS72 uid54507 |  |
|  |  | *Streptococcus pneumoniae* SP3 BS71 uid54493 |  |
|  |  | *Streptococcus pneumoniae* SP6 BS73 uid54495 |  |
|  |  | *Streptococcus pneumoniae* SP9 BS68 uid54497 |  |
|  |  | *Streptococcus pneumoniae* SP9v BS293 uid51547 |  |
| *Streptococcus porcinus* | 1 | *Streptococcus porcinus* Jelinkova 176 uid66741 | + |
| *Streptococcus pseudoporcinus* | 1 | *Streptococcus pseudoporcinus* SPIN 20026 uid61043 | + |
| *Streptococcus pyogenes* | 2 | *Streptococcus pyogenes* ATCC 10782 uid52353 |  |
|  |  | *Streptococcus pyogenes* M49 591 uid54147 |  |
| *Streptococcus salivarius* | 1 | *Streptococcus salivarius* SK126 uid55863 |  |
| *Streptococcus sanguinis* | 2 | *Streptococcus sanguinis* ATCC 49296 uid61461 | + |
|  |  | *Streptococcus sanguinis* VMC66 uid62785 |  |
| *Streptococcus vestibularis* | 2 | *Streptococcus vestibularis* ATCC 49124 uid62665 |  |
|  |  | *Streptococcus vestibularis* F0396 uid60559 | + |
