# Supplementary material for: Type-IVC Secretion System: A Novel Subclass of Type IV Secretion System (T4SS) Common Existing in Gram-Positive Genus Streptococcus
Source: PLoS One. 2012 Oct 4;7(10):e46390. doi: 10.1371/journal.pone.0046390 (PMC3464263; doi:10.1371/journal.pone.0046390)
Supplement: Protocol S1 — Detailed Material and Methods. (DOC) [file pone.0046390.s005.doc]

**Protocol S1. Detailed Materials and Methods**

The identification of VirB/D genes was performed via the following protocol:

1. On the basic of their similarity to the sequences of the COG database ^1^, all the genes of the downloaded genomes were annotated, and genes related to T4SS were selected as candidate VirB/D genes.
2. The protein sequences of genes in Streptococcus genomes were compared with 839 proven VirB/D genes using BLAST ^2^. If the genes could be aligned with proven VirB/D genes and their alignment sequences were larger than 50% of the query length with >=25% identity or >=40% positivity, they were considered candidate VirB/D genes.
3. Genes with the functional domain of VirB/D genes were predicted by the program of HMMER ^3^ with parameters (--domE 0.01 and -E 10) and considered candidate VirB/D genes. These functional domains were downloaded from PFAM database (<http://pfam.sanger.ac.uk/>).

Not every identified VirB/D was incorporated functionally in T4SS system. These functional VirB/D genes were usually clustered together in the genome^4^. On the basis of the locations of the predicted VirB/D genes in the genomes, GIs with VirB/D cluster in Streptococcus genomes were also identified. In this study, determination of a VirB/D cluster conformed to the following criteria: (1) the distance between two nearby VirB/D genes is less than 5kb, (2) the total length of the VirB/D cluster is less than 50kb, and (3) The number of VirB/D genes in a VirB/D cluster is >=3.

**Reference**

1. Tatusov, R. et al. The COG database: an updated version includes eukaryotes. *BMC Bioinformatics* **4**, 41 (2003).

2. Altschul, S.F., Gish, W., Miller, W., Myers, E.W. & Lipman, D.J. Basic local alignment search tool. *Journal of Molecular Biology* **215**, 403-410 (1990).

3. Eddy, S.R. Profile hidden Markov models. *Bioinformatics* **14**, 755-763 (1998).

4. Fronzes, R., Christie, P.J. & Waksman, G. The structural biology of type IV secretion systems. *Nat Rev Micro* **7**, 703-714 (2009).
